# The Weekly Day off

**Published:** 1920-05-15

**Authors:** 


### Transvaal Trained Nurses' Association.

### The Weekly Day Off.

Miss Ellershaw presided lately at a meeting of the Trained Nurses' Association, held at the General Hospital, Johannesburg.

Mr. H. J. Lamb, M.P.C., addressed the meeting as the person responsible for the Private Hospitals Ordinance passed by the Transvaal Provincial Council, which had been severely criticised. The primary object of the Ordinance was, he said, to put a stop to the overworking of nurses. While in hospital eight years ago he became convinced that seven days a week for four weeks at a stretch was bad for the health of young women. In 1916 the Transvaal Administrator appointed a Commission. The Commission took evidence at all the principal hospitals, from all grades of nurses, as well as from superintendents and matrons, with the result that there was a consensus in favour of a day off per week. The Nurses' Relief Ordinance was passed by the Provincial Council in the following year. Mr. Lamb said he became convinced that the hours of nurses in private hospitals were exorbitant, and as the Trained Nurses' Association had failed to limit these hours, he then, as a member of the Transvaal Provincial Executive, brought in the Private Hospitals Ordinance, which included clauses introducing the supervision of private nursing homes. He invited the co-operation of the T.N.A. to amend the law still further, if desirable; but forty-eight hours was considered sufficient for an able-bodied man, and, as the existing Ordinance allowed nurses to work fifty-four hours a week, they must effect a still further reduction of working hours. If elected to the next Provincial Council he would take in hand the question of the registration of nurses, and the making it a penal offence for any but a certificated nurse to wear hospital uniform.

Miss Elliot said that the nursing profession could not be compared with butchers, drapers, or accountants, who could stop work for a day or two without serious harm. In case of illness this could not be done by nurses. A constant change of nurses might constitute a real danger in certain surgical cases. She objected to the "six-nights" clause as likely to be harmful to the nurse.

Miss Jones thought that the Ordinance should state specifically that the matron had the right to grant or refuse a probationer twenty-four hours' leave.

Miss Winter pointed out that Government hospitals were often run at a loss. But private institutions could not do that. No doubt the Ordinance had been drawn up with the best intentions, but the Trained Nurses' Association should have been directly consulted. The rank and file, not being trained nurses, could not be members of the T.N.A. until they had qualified.

Mr. Lamb, in reply, said that it had been intended originally to provide that managers of nursing institutions should be registered trained nurses, but, as it had been represented that doctors themselves ran private nursing homes, the provision had been deleted.

Sister Alexander, in proposing a vote of thanks to Mr. Lamb, emphasised the necessity for the Provincial Executive co-operating with the T.N.A. in all future legislation. It was quite impossible to tie a nurse to eight hours a day. In England it had been found better to make a ninety-six-hour fortnight. Nurses quite appreciated one day off, but, owing to the want of provision for the conditions it brought about, their examination results had been most unsatisfactory.

